# Evaluation of Potential ARG Packaging by Two Environmental T7-Like Phage during Phage-Host Interaction

**DOI:** 10.3390/v12101060

**Published:** 2020-09-23

**Authors:** Junlin Liu, Peng Liu, Fenglin Feng, Junxuan Zhang, Fulin Li, Mianzhi Wang, Yongxue Sun

**Affiliations:** 1 National Risk Assessment Laboratory for Antimicrobial Resistance of Animal Original Bacteria, South China Agricultural University, Guangzhou 510640, China; liujunlin448@gmail.com (J.L.); fengfl0926@gmail.com (F.F.); zhangjx20615@gmail.com (J.Z.); fulinli570@gmail.com (F.L.); wangmz@yzu.edu.cn (M.W.); 2 Guangdong Provincial Key Laboratory of Veterinary Pharmaceutics Development and Safety Evaluation, College of Veterinary Medicine, South China Agricultural University, Guangzhou 510640, China; 3 Beijing Advanced Innovation Center for Food Nutrition and Human Health, College of Veterinary Medicine, China Agricultural University, Beijing 100193, China; liupeng_cau@163.com

**Keywords:** T7-like phage, antibiotic resistance genes (ARG), potential packaging, phage-host interaction

## Abstract

The increase in antimicrobial resistance is a threat to both human and animal health. The transfer of antibiotic resistance genes (ARG) via plasmids has been studied in detail whereas the contribution of bacteriophage-mediated ARG transmission is relatively little explored. We isolated and characterized two T7-like lytic bacteriophages that infected multidrug-resistant *Escherichia coli* hosts. The morphology and genomic analysis indicated that both phage HZP2 and HZ2R8 were evolutionarily related and their genomes did not encode ARGs. However, ARG-like raw reads were detected in offspring sequencing data with a different abundance level implying that potential ARG packaging had occurred. PCR results demonstrated that six fragments of genes (*qnrS*, *cmlA*, *tetM*, *bla_TEM_*, *sul3*, *mcr-1*) were potentially packaged by phage HZP2 and four (*qnrS*, *cmlA*, *bla_TEM_*, *mcr-1*) by phage HZ2R8. Further quantitative results showed that ARG abundance hierarchies were similar. The gene *bla_TEM_* was the most abundant (up to 1.38 × 10^7^ copies/mL) whereas *cmlA* and *qnrS* were the least. Moreover, the clinically important *mcr-1* gene was the second most abundant ARG indicating a possibility for spread through generalized transduction. Together, our results indicated that these structurally similar phage possessed similar characteristics and potential packaging during phage-host interaction displayed an ARG preference rather than occurring randomly.

## 1. Introduction

Antibiotics have been in use for almost a hundred years since penicillin was first reported in 1928 [1,2]. These drugs have made significant contributions to treatment of bacterial infections. However, antibiotic resistance among bacteria has become a major global health problem due to the overuse of antibiotics especially in the veterinary and medical fields [3]. As an important infectious pathogen of both humans and animals [4,5,6], multidrug-resistant *Escherichia coli* from farm animals may pose a zoonotic risk creating another layer of adverse health effects [7,8].

Bacteria acquire antibiotic resistance through chromosomal mutations and horizontal gene transfer (HGT). The latter processes primarily include transformation and conjugation although antibiotic resistance gene (ARG) transduction has also been demonstrated [9,10]. However, HGT processes mediated by transposons, integrons and plasmids through transformation and conjugation are better described. Less is known about the relative contribution that phage-mediated transduction plays in ARG transfer by HGT [11,12]. As natural bacterial killer, Bacteriophage (phage) infect their hosts and subsequently inject their genomes into the bacterial cells, after which one of two outcomes may occur. One has a lytic life cycle involving generalized transduction, and the other has a lysogenic life cycle involving generalized and specialized transduction [13]. In generalized transduction, during assembly of phage component to form progeny phage, sometime any of the fragments from donor DNA get incorporated into the phage capsid or DNA probably, including ARGs in bacterial chromosomes or plasmids (potential packaging). Then, such progeny phage are released and can re-infect hosts to disseminate these fragments at low frequencies [14]. In specialized transduction, only a few restricted genes (DNA fragments) from donor bacteria can be transferred by phage.

Generalized transduction generally yields 10^−6^ to 10^−7^ transductants/pfu implying that this process is infrequent [15]. However, although generalized transduction rare in any one replication cycle, phage are the most abundant biological entities in ecological environment, implying we cannot ignore the contribution of generalized transduction [14,16]. For example, our previous metagenomic analysis indicated a high abundance of ARGs encoding resistance to tetracyclines, aminoglycosides and macrolides in phage metagenomes taken from a sewage treatment system [2,17]. ARGs have also been detected in phage metagenomes from healthy human feces [18] as well as a relatively high-level ARG abundance in phage metagenomes from wastewater [19]. A single-cell analysis also indicated that erythromycin resistance genes encoded on a transposon could be transmitted by generalized transduction at an average frequency of 10^−6^ transductants/pfu [20]. ARGs have also been found phage DNA at levels as high as 10^3^ copies/mL wastewater [21].

The phenomenon of phage to harbor ARG has been revealed through metagenomic analyses. In this work we explored the ability of wild-type lytic phage to acquire ARGs at the single cell level. We isolated and characterized two phage from a pig-derived sewage treatment system. Potential packaging of ARGs during phage-host interaction was further explored using genome sequencing and quantitative PCR (qPCR). 

## 2. Materials and Methods

### 2.1. Bacterial and Phage Strains

In this study we utilized 135 *Escherichia coli* strains that had been previously isolated from pig feces and were identified using matrix-assisted laser desorption ionization–time of flight mass spectrometry (MALDI-TOF-MS) [22]. Strains HZA135 and HZA50 identified in this group were the phage hosts used in the current study. Minimum inhibitory concentrations (MIC) for commonly used antibiotics were determined using the broth dilution method [23], then ARGs carried by bacteria further were verified with PCR method. Strain HZA135 was resistant to ampicillin, amoxicillin, colistin, neomycin, doxycycline, tigecycline, florfenicol and sulfamethoxazole. This strain also possessed the ARGs *ermB*, *floR*, *bla_TEM_*, *tetM*, *sul1*, *qnrS*, *cmlA* and *mcr-1*. Strain HZA50 was resistant to ampicillin, amoxicillin, colistin, florfenicol and possessed the ARGs *floR*, *bla_TEM_*, *qnrS*, *cmlA* and *mcr-1* (Supplementary Materials Table S1).

Phage were isolated from wastewater samples as previously described [21]. In brief, wastewater samples from the pig farm sewage treatment system were centrifuged to remove solids and the supernatant was filtered through a 0.22-μm pore membrane. The filtrate was combined separately with logarithmic phase cells of 135 *E. coli* strains and incubated with shaking overnight at 37 °C. The mixed culture was again centrifuged and filtered as per above and used to inoculate new bacterial cultures and the crude phage preparation were repeated. The final filtrates were then plaque-purified by culture in 5 mL LB containing 0.7% agar overnight and large, clear and round plaques were selected and diluted in SM buffer (100 mM NaCl, 8 mM MgSO_4_·7H_2_O, 50 mM Tris-HCl, pH = 7.5) and stored at 4 °C. Plaque purification was repeated at least three additional times to ensure single phage were isolated.

### 2.2. Transmission Electron Microscopy (TEM)

Plaque-purified phage were added to a copper grid and allowed to attach for 15 min. The samples were then negatively stained with 2% phosphotungstic acid as previously described [24]. Phage morphology was observed using a Tecnai 12 transmission electron microscope (FEI Thermo Fisher, Hillsboro, OR, USA) at 80 kV.

### 2.3. Phage Testing Procedures

Phage thermostability was examined by incubating phage filtrates at 4, 37, 50, 55, 60, and 70 °C for 60 min. The pH stability was examined by incubating filtrates over a pH range of 1 to 12 for 24 h at 37 °C as previously described with a few modifications [25]. Phage titers were assayed by plating in soft agar (see above) and each test was performed in triplicate.

Phage host ranges were determined by examining plaque formation in bacterial lawns of 135 *E. coli* strains used as target cells as described previously [26]. The multiplicity of infection (MOI) was examined using appropriate susceptible *E. coli* strains cultured to early log phase growth and combined with phage (P:B = 10:1, 1:1, 0.1:1, 0.01:1, 0.001:1 and 0.0001:1) using standard protocols in triplicate. 

One-step growth experiments were carried out in triplicate as described previously with a few modifications [27]. In brief, the host *E. coli* strain was grown until early log phase and phage were added at an MOI of 10 and allowed to attach for 10 min at room temperature, the cells were centrifuged and the supernatant was discarded and infected cells were suspended in LB medium and incubated with shaking at 37 °C. Samples were obtained at 10 min intervals to determine phage titers. Burst sizes were calculated by dividing the phage titers at plateau phase by the initial concentration of infected bacterial cells.

### 2.4. Genome Analysis

Total bacterial DNA was extracted using a commercial kit (Water DNA Kit) using the recommendations of the manufacturer (Omega Bio-Tek, Norcross, GA, USA). The purified phage lysates were treated with DNase I and RNase A both at 1 µg/mL (Sangon Biotech, Shanghai, China) before DNA extraction. Phage DNA was extracted as described previously [28]. DNA precipitates were solubilized in TE (10 mM Tris–HCl, pH 7.5, 1 mM EDTA) buffer and DNA concentrations were determined using UV spectroscopy with an ND-1000 instrument (NanoDrop, Wilmington, NC, USA). Purified DNA samples were commercially sequenced at Genewiz (Guangzhou, China) using the Illumina Hiseq 4000 platform. Poor quality reads were filtered from raw sequencing data to obtain clean reads that were then assembled into complete genomes using Velvet (version 1.2.10), SSPACE (version 3.0) and GapFiller software (version 1.10). 

The raw sequencing data was used for ARG identification and annotation and were then grouped according to the mechanism of antibiotic resistance [29]. The requirements for being grouped as an ARG-like fragment used a cutoff value of 10^−7^, sequence identity of ≥90% and an alignment length of >25 amino acids [30,31,32]. 

Open reading frames (ORF) of phage complete genome sequences were predicted using orffinder (https://www.ncbi.nlm.nih.gov/orffinder/). BLASTP (https://blast.ncbi.nlm.nih.gov/) was used to search non-redundant protein sequence databases and optimal matches were selected after comparing the similarity and identity of proteins with known functions (*E* value of the sequencing alignment was 10^−5^). tRNA-encoding genes, virulence factor and antibiotic resistance genes were detected using the tRNAscan-SE database [33], the online website VFDB (virulence factor database) and CARD (antibiotic resistance comprehensive database), respectively. The complete genome map and GC shift of the phages were drawn through the online website CGview Server (http://cgview.ca/). The phage genomes were compared using BLASTN (https://blast.ncbi.nlm.nih.gov/) and an image was drawn using Easyfig (version 2.2.2) after alignment to related phage genomes. Phylogenetic analyses between the related phage genomes were carried out by comparing large terminase subunit and T7 DNA polymerase using the program ClustalW in MEGA (Version 6.0) [34]. Phylogenetic trees were constructed with MEGA using the Neighbor-Joining algorithm.

### 2.5. Potential ARG Packaging

We used 4 phage enrichment procedures using the following bacterial concentrations (CFU/mL) and MOI values: G1, 10^8^ and 10^3^; G2, 10^8^ and 10; G3, 10^5^ and 10^3^; G4, 10^5^ and 10. Phage were pretreated with 1 µg/mL DNase I (see above) and phage lysates were concentrated to 0.25 mL by centrifugation using 100 kDa cutoff Amicon (Millipore Sigma, Burlington, MA, USA) ultrafiltration tubes. Extraction of phage DNA using a commercial kit (Viral DNA Kit) using the conditions suggested by the manufacturer (Omega Bio-Tek, Norcross, GA, USA) avoided DNA loss.

PCR reactions (see below) were carried out in triplicate as described previously (Table S2) [17]. Amplicons generated using primers for seven genes (*ermB*, *qnrS*, *cmlA*, *tetM*, *bla_TEM_*, *sul3*, *mcr-1*) and *16s rDNA* were purified from agarose gels using a Poly-Gel DNA Extraction Kit (Omega) and ligated into the plasmid vector pMD19-T vector (Takara Biotech, Dalian, China). Recombinant plasmid vectors were amplified in the bacterial host *E. coli* DH5α (TransGen Biotech, Beijing, China) and plasmid DNA was extracted using the Plasmid DNA Kit (Omega Bio-Tek, Norcross, GA, USA) and commercially sequenced by Sangon Biotech (Shanghai, China) (Table S3).

Real time quantitative PCR (qPCR) reactions contained 10 µL SYBR Premix Mix Ex Taq II (Takara Biotech, Dalian, China), 0.8 µL each primer, 1 µL DNA template in a total volume of 20 µL. Amplification was carried our using a Bio-Rad Chromo4 instrument (Biorad, Hercules, CA, USA) equipped with the data analysis software Bio-Rad CFX Manager (version 3.1). The amplification conditions were 95 °C for 3 min, followed by 40 cycles of 95 °C for 15 s, annealing for 45 s and an extension at 72 °C for 1 min. Negative controls of nuclease-free water were paired with each sample. The qPCR efficiency was maintained at 90–120% and R^2^ values for calibration curves were >99.0%. 

### 2.6. Data Analysis

Significant changes in ARG gene abundance were analyzed using SPSS version 25 (IBM, Chicago, IL, USA).

### 2.7. Intact ARG Testing

Intact target ARGs were detected in phage DNA by PCR (Table S4). PCR products covered over 90% of intact target ARGs. The amplification conditions were 95 °C for 5 min, followed by 30 cycles of 95 °C for 45 s, annealing for 45 s and an extension at 72 °C for 90 s. Negative controls of nuclease-free water were paired with each sample.

## 3. Results

### 3.1. Phage Characterization

**Figure 1:**
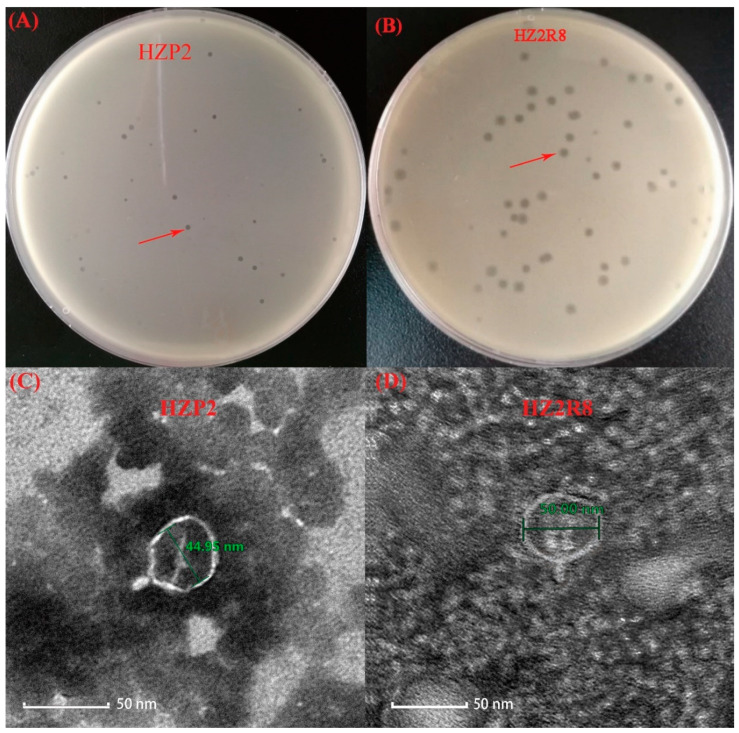
Isolated of phage HZP2 and HZ2R8. (**A**) Plaque morphology of phage HZP2. (**B**) Plaque morphology of phage HZ2R8. (**C**) Transmission electron microscopy of phage HZP2, where the scale bar represented 100 nm. (**D**) Transmission electron microscopy of phage HZ2R8, where the scale bar represented 50 nm.

We identified wild-type phage HZP2 infecting *E. coli* host HZA135 and wild-type phage HZ2R8 infecting *E. coli* host HZA50, respectively. Both HZP2 and HZ2R8 formed clear round plaques of 1 and 2 mm diameters, respectively (Figure 1A,B). The ultrastructure of both these phage revealed they were *Podoviridae* family members and linear dsDNA phage characterized by the presence of very short, non-contractile tails [35]. The average capsid head diameter of phage HZP2 was 44.95 nm and it possessed a short, stubby tail of about 10 nm in length (Figure 1C). Phage HZ2R8 possessed an average capsid head diameter of 55 nm and also possessed a 10 nm tail (Figure 1D). 

**Figure 2:**
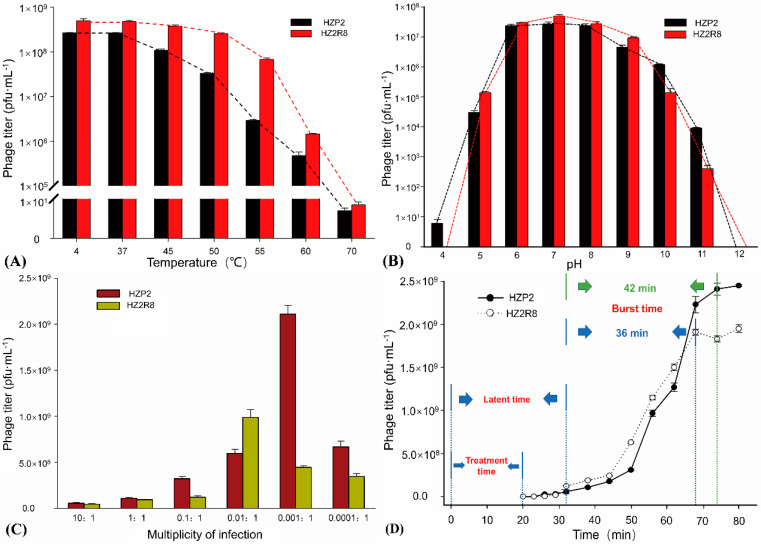
General features of phage HZP2 and HZ2R8. (**A**) Thermal stability test of phage HZP2 and HZ2R8. (**B**) pH stability test of phage HZP2 and HZ2R8. (**C**) Multiplicity of infection (MOI) experiments of phage HZP2 and HZ2R8. (**D**) One step-growth cure of phage HZP2 and HZ2R8.

Both phage HZP2 and HZ2R8 were stable at 4 and 37 °C and the titers gradually decreased at temperatures between 50 and 60 °C. Phage plaques were almost absent after treatment at 70 °C (Figure 2A). The pH stability tests indicated that phage HZP2 was stable at pH 6–10. However, at pH 5 or 11 the titers were reduced by about three orders of magnitude and in the pH range of 1–4 or at pH 12, HZP2 was nearly inactive. The phage HZ2R8 was stable at pH 6–9 and at pH of 5 or 10 the titers decreased by about three orders of magnitude and five orders of magnitude at pH 11. After incubation at pH values from 1–4 or at pH 12, phage HZ2R8 was inactive (Figure 2B).

The host ranges for phage HZP2 and HZP2R8 were different except both could infect strain A-107. Phage HZP2 could lyse (formed clear plaques) on *E. coli* strains A-13, A-24, A-29, A-107 and A-135 (3.7%). Phage HZ2R8 could lyse strains A-5, A-16, A-45, A-46, A-50, A-58, A-59, A-70, A-74, A-96, A-104, A-107, A-114 and A-120 (10.37%). The titers of phage HZP2 were the greatest at 2.11 × 10^9^ pfu/mL when using an MOI of 0.001. The titers of phage HZ2R8 were the greatest at 9.89 × 10^8^ pfu/mL using an MOI of 0.01 (Figure 2C). A one-step growth curve showed that attachment and cell penetration times were 20 min for both phage with a latent period of 32 min. The burst times for HZP2 and HZ2R8 were 42 and 36 min, respectively. The calculated burst sizes were 120 and 93 pfu/cell, respectively (Figure 2D).

### 3.2. Genome Annotation and Characteristics

**Figure 3:**
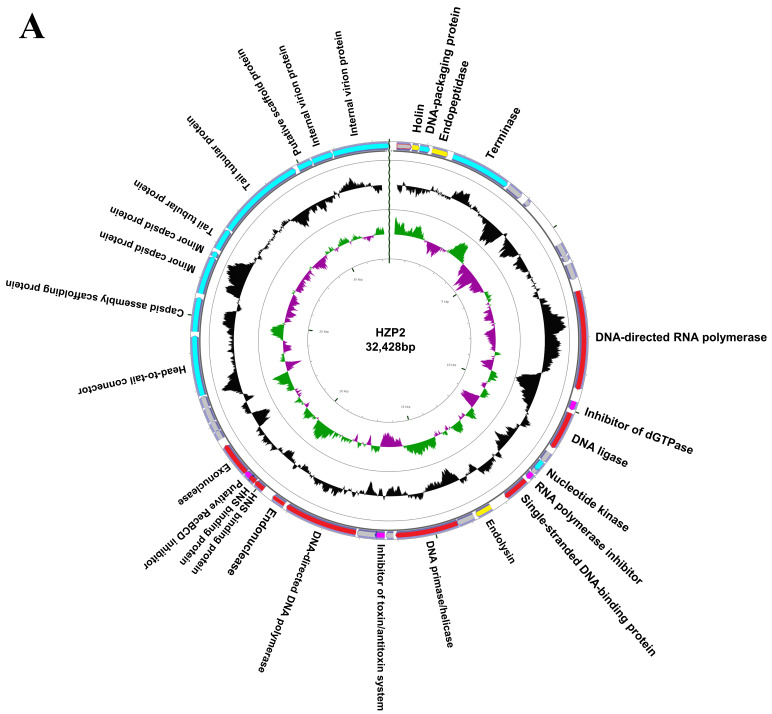

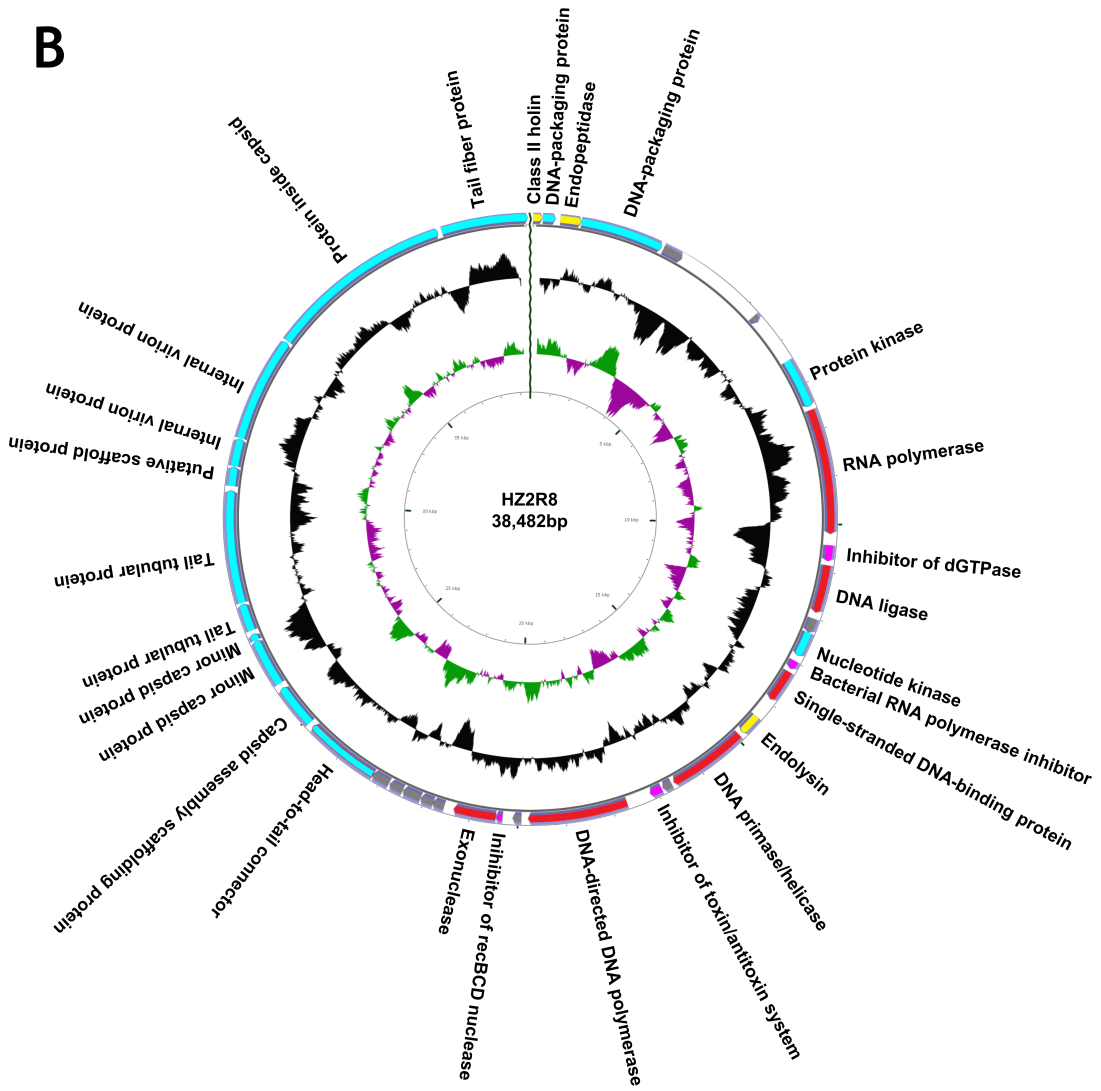
Map of the genome organization of phage HZP2 (**A**) and HZ2R8 (**B**). Different colors in the first circle represented putative open reading frames (ORFs) with different functions. Red represented DNA replication and regulation modules, blue represented packaging and structural modules, yellow represented lysis modules, pink represented host function inhibition modules, and gray represented hypothetical proteins modules. The second circle showed the G/C content: outward indicated that the G/C content of the region was higher than the average G/C content of the whole genome, and inward indicated that G/C content of the region was less than the average; The third circle showed the GC skew. Green outward indicated the area G > C, and pink inward indicated G < C.

**Figure 4:**
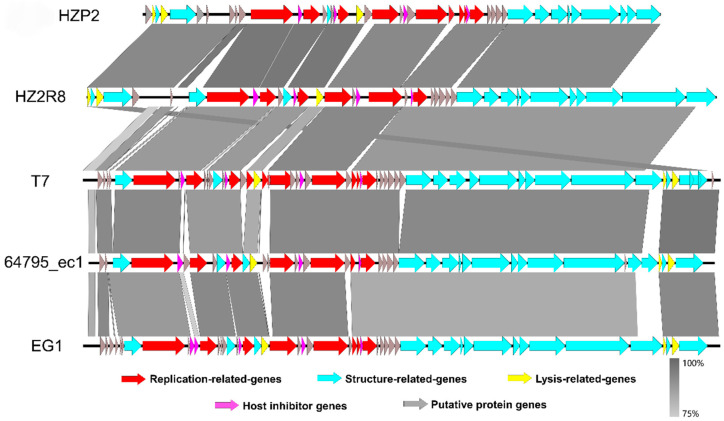
ORF-by-ORF comparison of the genomes of phage HZP2 and HZ2R8 and other homologous phages. The functional modules indicated by color. Similarities were showed in gray according to the scale on the left side.

We completely sequenced the genomes of both phage and both contained linear, double stranded genomes. Phage HZP2 was 32,448 bp in length with a 48.30% GC content and 43 putative open reading frames (ORF). These 43 predicted functional proteins could be categorized into five functional groups: 9 DNA replication and regulation modules, 12 packaging and structural modules, 3 host lysis modules, 4 host function inhibition modules and 15 hypothetical protein modules (Figure 3A). Phage HZ2R8 was 39,482 bp in length with a 48.61% GC content and 38 putative ORFs. The 38 predicted functional proteins were categorized into five functional groups: 6 DNA replication and regulation modules, 15 packaging and structural modules, 3 host lysis modules, 4 host function inhibition modules and 10 hypothetical protein modules (Figure 3B). Phage HZP2 and HZ2R8 hypothetical protein modules were similar (Figure 4). We also compared and annotated each phage (Tables S5 and S6) and their sequences were submitted to GenBank under accession numbers MK542821 and MG832642, respectively.

When we analyzed the complete genome sequences for the phage in which we did not detect tRNA, virulence factor genes or ARGs. However, 10,344 paired reads aligned to target ARGs in the raw sequencing data of HZA135 and included fragments encoding resistance in 2393 to β-lactams (1492 *bla_TEM_*), in 1961 to tetracyclines (754 *tetM*), in 1951 to colistin (1949 *mcr-1*), in 2362 to phenicols (1052 *cmlA*), 952 to quinolones (653 *qnrS*) and 725 to sulfonamides (667 *sul3*). Moreover, 302 paired reads aligned to target ARGs in raw sequencing data of phage HZP2 included those encoding resistance of 73 to β-lactams (58 *bla_TEM_*), 177 to tetracyclines (106 *tetM*), 18 to colistin (18 *mcr-1*), 12 to phenicols (6 *cmlA*), 1 to quinolones (*qnrS*) and 21 to sulfonamides (21 *sul3*).

From the results of HZP2 genome comparison using BLASTN, four T7-like *E. coil* phages with higher total scores (>80% coverage, >85% identity) were selected from different regions around the world (Table S7). Although the coverage of phage EG1 genome was the highest, the structural similarities between phage HZP2 genome and phage HZ2R8 genome was the most similar. Differed for phage T7, 64795_ec1 and EG1 genome structure, the distribution of lysis modules in HZP2 and HZ2R8 was atypical. The length of the packaging and structural modules in phage HZP2 were the shortest of these phage (Figure 4).

**Figure 5:**
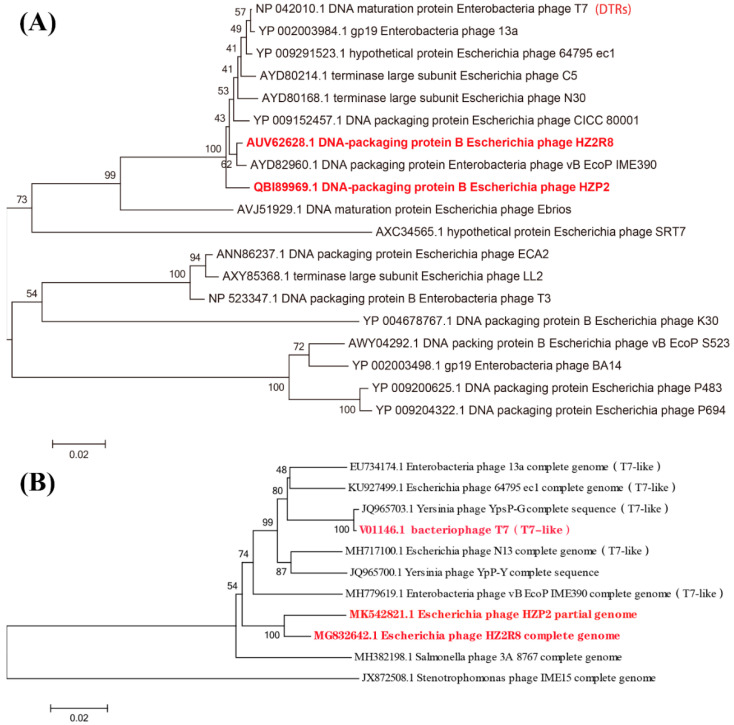
(**A**) Phylogenetic tree of various phages based on large terminase subunit. (**B**) Phylogenetic tree of various phages based on DNA polymerase.

The large terminase subunit of HZP2 was most closely related to phage vB EcoP IME390 and then HZ2R8; the latter was most closely related to phage vB EcoP IME390 and then CICC 80001, HZP2 and N30 (Figure 5A). The DNA polymerase of HZP2 and HZ2R8 were closely related and formed a clade with T7-like phage (Figure 5B).

### 3.3. Potential ARG Packaging

**Figure 6:**
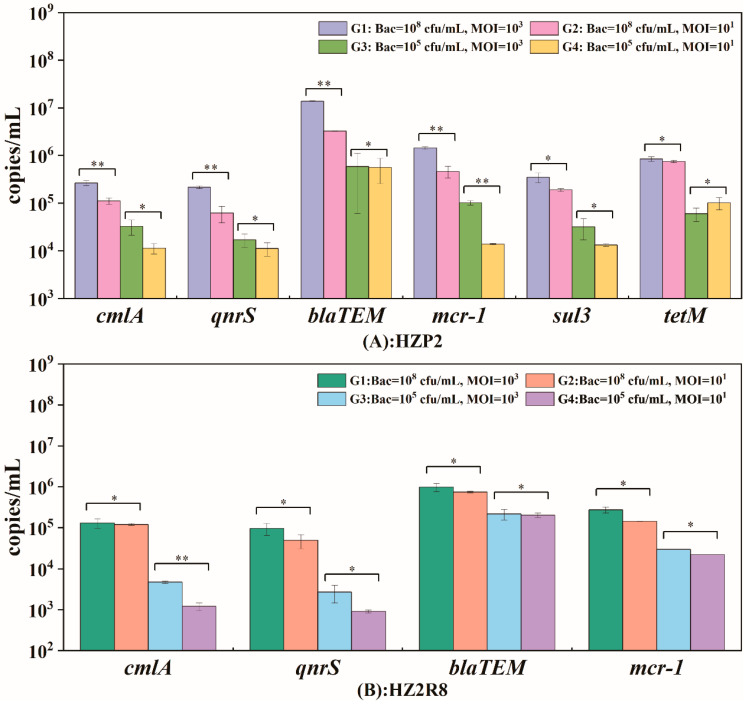
The potential packaging of antibiotic resistance genes (ARGs) in phage HZP2 (**A**) and HZ2R8 (**B**). Data are from three independent cultures, and one standard deviation is shown. Significant changes were marked with two asterisks for *p* < 0.05, otherwise marked with one asterisk.

We analyzed the ARG gene abundance for phage HZP2 and HZ2R8 and 6 genes were present in HZP2 DNA (*qnrS*, *cmlA*, *tetM*, *bla_TEM_*, *sul3*, *mcr-1*) and 4 (*qnrS*, *cmlA*, *bla_TEM_*, *mcr-1*) in phage HZ2R8 DNA. Quantitatively, the ARG gene abundance of phage HZP2 and HZP2R8 presented similar fixed trends (Figure 6). The fixed hierarchy for HZP2 was *bla_TEM_* > *tetM* ≈ *mcr-1* > *sul3* > *cmlA* > *qnrS* and for HZP2R8 was *bla_TEM_* > *mcr-1* > *cmlA* > *qnrS*.

We examined whether the host cell concentrations and MOI were factors in determining absolute ARG abundance. The group G1 contained the most ARGs for both HZP2 and HZ2R8 and at 2.8 × 10^6^ and 3.7 × 10^5^ copies/mL, respectively. The ARG *bla_TEM_* was the most abundant overall at 1.38 × 10^7^ copies/mL in phage HZP2 while the lowest was *qnrS* at 2.17 × 10^5^ copies/mL. The most abundant ARG in HZ2R8 was *bla_TEM_* at 9.84 × 10^5^ copies/mL and the lowest was *qnrS* with 9.63 × 10^4^ copies/mL. In the G2, G3 and G4 groups, ARG gene abundance was 8.0 × 10^5^, 1.4 × 10^5^ and 1.2 × 10^5^ copies/mL, respectively for phage HZP2. For HZ2R8, ARG abundance was 2.6 × 10^5^ and 6.4 × 10^4^ and 5.7 × 10^4^ copies/mL, respectively. Increasing the MOI by two orders of magnitude (G3 and G4) using 10^5^ host cells the ARG gene abundance for phage HZP2 was not significantly altered with the exception of *mcr-1*. When the host levels were 10^8^ cells/mL (G1 and G2), *cmlA*, *qnrS*, *bla_TEM_* and *mcr-1* were significantly altered. However, in phage HZ2R8 DNA, host bacteria levels of 10^5^ or 10^8^ did not significantly alter ARG gene abundance with the exception of *cmlA*.

We also detected the intact target ARGs in phage DNA by PCR method. All results were negative (Table 1).

## 4. Discussion

Increasing antimicrobial resistance among bacteria poses a serious threat to human and animal health and public environmental safety due to overuse of antibiotic [36]. Bacteria can acquire ARGs through HGT including phage transduction [37,38,39,40], however, the roles that phage play in ARG acquisition has not been well characterized during phage-host interaction.

In this study, we isolated two phage (HZP2 and HZ2R8) using two *E. coli* strains isolated from pig wastewater. Both phage formed clear plaques of 1–2 mm and were *Podoviridae* family members [41]. The morphologies of phage HZP2 and HZ2R8 closely resembled the T7-like phage CICC 80001 [42] and vB_EcoP-EG1 [43], suggesting these are T7-like phage. Both phage were thermally and pH stable and both had limited host ranges. However, phage HZP2 lysed cells more rapidly at an MOI of 0.001 and HZ2R8 at 0.01. One-step growth experiments were then conducted using an MOI of 10 to ensure infection of the total bacterial population. The burst size of phage HZP2 was about 120 and HZ2R8 was 93.

The genomes of both phage were linear dsDNA molecules about 32.5 and 39.5 kbp in length containing about 48% GC. Significantly, no tRNA or virulence genes or ARGs were present in either phage indicating they are potential candidates for phage therapy. The overall genomic structures of HZP2 and HZ2R8 genome were similar although their modular distribution was atypical. Together, these results indicated that phage HZP2 and HZP2R8 were similar and novel phage.

Phage terminal protein, which was divided into large and small subunits according to molecular weight, was one of the few necessary functional proteins in the assembly process of dsDNA virus replication and determined phage packaging mechanism [44]. The terminase large subunit was used to confirm the phylogenetic analysis after each phage was assigned a taxonomical lineage up to the genus level. These results demonstrated a close evolutionary relationship between HZP2 and HZ2R8 at the genus level and both the packaging mechanism of them might be direct terminal repeats (DTRs) [45]. The DNA polymerase of T7-like phages is also a conserved protein often used to study the global distribution and diversity of phage in a manner analogous to the bacterial 16S rRNA [46]. The DNA polymerase of HZP2 showed 99% identity to T7-like phage while HZ2R8 showed 99% identity to T7 and 64795_ec1. These results indicated that HZP2 and HZ2R8 are T7-like phage members. In contrast to the latter, HZP2 did not contain the typical full complement of internal and tail fiber proteins and may reflect differences as is common in the T7-like group such as for phage T7, Salmonella phage P22 and Vibrio parahaemolyticus phage OWB [47,48,49]. However, this also suggests that the absorption machinery of HZP2 may differ from other T7-like phage and limit its host range.

During generalized transduction, fragments of host DNA or plasmids are acquired by lytic phage though potential packaging. Only when the ARG fragment is packaged intact, the offspring phage have the potential to promote ARG transfer. We found that although the genomes of HZP2 and HZ2R8 did not contain ARGs, a subpopulation could be identified that did. That was because there were few fragments of ARGs in the phage genome, which were removed easily as foreign contamination. Meanwhile, we ruled out that contamination of foreign DNA after phage lysis by using DNase and RNase pretreatment steps and that was verified by 16S rDNA in phage DNA. Moreover, we analyzed whether the ARG fractions were associated with the host carrying ARGs and analyzed the raw sequencing reads of HZP2 infecting multidrug-resistant strain HZA135. We also compared whether host cell and phage concentrations altered levels of ARG gene abundance, and analyzed whether phage potentially packaged intact ARGs. Here, we found the phage HZP2 DNA contained 302 paired reads that aligned to host target ARGs. From the PCR and qPCR results, six genes (*qnrS*, *cmlA*, *tetM*, *bla_TEM_*, *sul3*, *mcr-1*) in phage HZP2 DNA and four genes (*qnrS*, *cmlA*, *bla_TEM_*, *mcr-1*) in phage HZ2R8 DNA were detected, indicating that the during phage-host interaction process, progeny phage could acquire host ARGs through potential packaging. We further tested the integrity of target ARGs in the phage DNA and found that all target ARGs could not be detected in phage DNA. These results suggested intact ARG fragments from phage DNA could be an important reservoir of ARGs in wastewater, which further confirmed our previously study [2].

In some cases, the specialized transduction frequencies of Staphylococcus aureus phage can reach 10^−1^ transductants/pfu due to the presence of mobile genetic elements [50], The Tn3-like transposase and serine recombinase genes of the *bla_TEM_* resistance gene also were found in temperate phage DNA such that *bla_TEM_* could be transferred using a typical transposase-like mechanism [51]. Our raw sequence analysis indicated the aligned reads of *bla_TEM_* was in high level both in HZP2 and HZA135. The qPCR results indicated that ARGs were detected in progeny phage at different abundance levels and the fragments of *bla_TEM_* gene was highest both in phage HZP2 and HZ2R8 DNA. In the G1 group we calculated the phage copies/pfu between gene abundance and phage concentration and found that *bla_TEM_* for phage HZP2 was 1.38 × 10^−4^ copies/pfu while for HZ2R8 was 9.84 × 10^−6^ copies/pfu. This indicated that one fragment of *bla_TEM_* gene was packaged potentially for every 7247 HZP2 and 101678 HZP2R8 progeny. These results suggested that although the strategy of generalized transduction differed from specialized transduction, the fragments of *bla_TEM_* gene still could be packaged potentially and efficiently.

Metagenomic analyses showed a relative high abundance of genes encoding ribosomal protection protein in tetracycline resistance genes group was detected in the human feces and pig-derived sewage treatment system [2,52]. This indicated that phage are more likely to harbor ABC transporter family and ribosomal protection genes than other ARG types. Our single-cell analysis of aligned reads and qPCR results indicated that *tetM* abundance was great, implying offspring phage carrying the fragments of gene of tetracycline resistance genes group were greater possible to participate in transfer and further confirmed our previous study [2]. Gene *mcr-1* is a serious matter especially in multidrug-resistant gram-negative bacteria that produced carbapenemase [53]. Although that was the first report that the fragments of *mcr-1* were in offspring phage DNA, the gene abundance of *mcr-1* was not as high. More importantly, that further proved that the transfer of mcr-1 is not only related to plasmids, but also that phage have the potential to participate in. Overall, we further evaluated the role of phage in ARG acquisition during phage-host interactions. Our results indicated that potential packaging exhibits an ARG fragments preference rather than occurring randomly. This is most likely the result of the host genome environment, and this to be true for *bla_TEM_*, *tetM*. Meanwhile, as an important ARG, *mcr-1* did not be ignored. Although intact target ARGs did not exist in phage DNA, high gene abundance implied that phage still had potential to participate in ARG transfer.

We varied the concentrations of host bacteria and phage as a variable and the significant ARG changes for HZP2 was 41.7% (5/12) and for HZ2R8 12.5% (1/8). This implied that the concentration of phage HZP2 could affect potential packaging for different ARGs while this was not the case for HZ2R8. HZP2 affected the potential packaging process inefficiently at low host cell densities. We have guessed that bacterial populations coordinate their behavior to make them prone to viral predation and phage-host interaction as cell density increases using quorum sensing signaling [54]. This warrants further exploration. These results illustrated that environmental factors can alter the potential packaging process but not in highly significant ways.

## 5. Conclusions

In this study we isolated two wild-type phage (HZP2 and HZ2R8) from a pig farm sewage treatment system and characterized the level of potential packaging of host ARGs during phage-host interaction. These phage were *Podoviridae* family members with stable biological characteristics and evolutionarily related at the genus level and novel T7-like phage family members. These lytic phage were also ARG reservoirs constructed through potential packaging and this process was affected by ARG type and phage concentration. The genes *bla_TEM_*, *tetM* and *mcr-1* would be a focus in future research. Our results indicated that these lytic phage have potential to participate in ARG transfer during generalized transduction.

## Figures and Tables

**Table 1:** Target ARG results of hosts and phage.

Gene	Size (bp)	HZA135	HZA50	HZP2	HZ2R8
*bla_TEM_*	247(28.7%)	+	+	+	+
	752(87.3%)	+	+	−	−
*qnrS*	428 (65.1%)	+	+	+	+
	619 (94.2%)	+	+	−	−
*cmlA*	158 (12.5%)	+	+	+	+
	1188 (94.3%)	+	+	−	−
*mcr-1*	220 (13.5%)	+	+	+	+
	1495 (92.0%)	+	+	−	−
*tetM*	171 (8.9%)	+	+	+	−
	1861 (96.9%)	+	−	−	−
*sul3*	128 (16.1%)	+	−	+	−
	714 (90.3%)	+	−	−	−
*ermB*	189 (25.6%)	+	−	−	−

+: Positive. −: Negative. The content in brackets represents the percentage to full-length gene.

## Supplementary Materials

The following are available online at https://www.mdpi.com/1999-4915/12/10/1060/s1, Table S1: Minimum inhibitory concentration testing of host bacteria. Table S2: PCR primers of fragment of genes used in this study. Table S3: Sequence comparisons of target ARGs. Table S4: PCR primers of intact genes used in this study. Table S5: ORF comparisons and annotations for phage HZP2. Table S6: ORF comparisons and annotations for phage HZ2R8. Table S7: Three T7-like *E. coli* phages with high total scores.
