# Supplementary material for: Evaluation of Potential ARG Packaging by Two Environmental T7-Like Phage during Phage-Host Interaction
Source: Viruses. 2020 Sep 23;12(10):1060. doi: 10.3390/v12101060 (PMC7598189; doi:10.3390/v12101060)
Supplement: Supplementary file 1 [file viruses-12-01060-s001.pdf]

Supplementary materials

Table S1: Minimum inhibitory concentration testing of host bacteria

Antimicrobial Agent	AMP	AMO	NEO	DOX	TIG	FLR	CL	SXT	CEF	CTX	CAZ	MEM	GEN	AMI	APR	CIP
S	≤8	≤8	≤4	≤4	≤1	≤2	≤2	≤2	≤2	≤1	≤4	≤1	≤4	≤16	≤8	≤1
I	16	16	8	8	2	4	-	-	4	2	8	2	8	32	16	2
R	≥32	≥32	≥16	≥16	≥4	≥32	≥4	≥4	≥8	≥4	≥16	≥4	≥16	≥64	≥32	≥4
HZA50	>128(R)	>64(R)	2(S)	1(S)	0.5(S)	64(R)	4(R)	2(S)	0.5(S)	0.125(S)	0.25(S)	0.016(S)	2(S)	2(S)	4(S)	1(S)
HZA135	>128(R)	>64(R)	64(R)	16(R)	4(R)	>128(R)	4(R)	>64(R)	0.5(S)	0.03(S)	0.25(S)	0.016(S)	1(S)	2(S)	8(S)	0.25(S)

Minimum inhibitory concentration (MIC) testing was conducted according to the recommendations of the CLSI2018. AMP: ampicillin; AMO: amoxicillin; NEO: neomycin; DOX: doxycycline; TIG: tigecycline; FLR: florfenicol; CL: colistin; SXT: trimethoprim-sulphamethoxazole; CEF: ceftiofur; CTX: cefotaxime; CAZ: ceftazidime; MEM: meropenem; GEN: gentamicin; AMI: amikacin; APR: apramycin; CIP: ciprofloxacin; R: resistant; I: intermaediate; S: susceptible.

Tables S2: PCR primers of fragments of gene used in this study

Gene		Primer (5'-3')	Size (bp)	Annealing temperature(°C)	References
sul3	F	TCCGTTCAGCGAATTGGTGCAG	128	60	（Pei et al., 2006）
	R	TTCGTTCACGCCTTACACCAGC			
qnrS	F	GCAAGTTCATTGAACAGGGT	428	54	（Cattoir et al., 2007）
	R	TCTAAACCGTCGAGTTCGGCG			
tetM	F	ACAGAAAGCTTATTATATAAC	171	45	（Aminov et al., 2001）
	R	TGGCGTGTCTATGATGTTCAC			
ermB	F	CCGTGCGTCTGACATCTATCT	189	56.8	（Guo et al., 2013）
	R	GTGGTATGGCGGGTAAGTTTT			
cmlA	F	GCCAGCAGTGCCGTTTAT	158	55	（Li et al., 2013）
	R	GGCCACCTCCCAGTAGAA			
mcr-1	F	TCAGCGATCATGGCGAAAGT	220	58.3	In this study

	R	CGGTCTTTGACTTTGTCCGC			
bla_{TEM}	F	GCKGCCAACTTACTTCTGACAACG	247	61	（Xi et al., 2009）
	R	CTTTATCCGCCTCCATCCAGTCTA			
16S rDNA	F	AAGAGTTTGATCCTGGCTCAGA	1503	61.7	（Calero-Cáceres et al., 2014）
	R	TACGGCTACCTTGTTACGACTT			

F: Forward primer. R: Reverse primer. The content in brackets refers to the size of entire gene.

Table S3: Sequence comparisons of target ARGs

Gene	Size(bp)	Accession	Match rate
tetM	171	KJ755874.1	100%
qnrS	428	KJ920352.1	100%
cmlA	158	CP036168.1	100%
sul3	128	MH765653.1	100%
mcr-1	220	MK256965.1	100%
bla_{TEM}	247	NC003486.1	100%
ermB	189	KX687895.1	99%

Tables S4: PCR primers of intact genes used in this study

Gene		Primer (5'-3')	Size (bp)	Annealing temperature(°C)	References
sul3	F	CGATAGTTTTTCCGATGGAGG	714	55.3	In this study
	R	TTAACATCATGGGTGCGGA			
qnrS	F	ATCGGCACCACAACTTTTC	619	52.7	In this study
	R	ACAATACCCAGTGCTTCGAG			
tetM	F	TTAGCTCATGTTGATGCAGG	1861	58.1	In this study
	R	CTATCCGACTATTTGGACGAC			

cmlA	F	ATCACCGTTCGATTTGCTG	1188	55.3	In this study
	R	GACGTACTTTCCGCACTTTG			
mcr-1	F	CAAACCTATCCCATCGCGGA	1495	52.7	In this study
	R	CGGTGCGGTCTTTGACTTTG			
bla_{TEM}	F	ACCCAGAAACGCTGGTGAAA	752	55.3	In this study
	R	TGAGGCACCTATCTCAGCGA			

F: Forward primer. R: Reverse primer.

Table S5: ORF comparison and annotation of phage HZP2

ORF	Start	Stop	Description	ID	Query cover	Evalue	Identify
1	194	598	hypothetical protein	AYD80164.1	99%	$1e^{-19}$	41.35%
2	608	811	holin class II	YP_009198335.1	100%	$1e^{-39}$	97.01%
3	811	1086	Terminase small subunit	AYD80166.1	100%	$3e^{-51}$	90.11%
4	1146	1586	endopeptidase	ATW57758.1	100%	$5e^{-91}$	89.73%
5	1719	3356	terminase large subunit	AUV62628.1	100%	0.0	98.90%
6	3394	3837	hypothetical protein	AUV62629.1	100%	$6e^{-105}$	99.32%
7	4003	4152	hypothetical protein	AAP34172.1	100%	$6e^{-23}$	91.84%
8	5444	5806	hypothetical protein	AFK13391.1	95%	$4e^{-69}$	92.11%
9	5806	5961	hypothetical protein	ATW57710.1	100%	$6e^{-30}$	100%
10	6024	6467	hypothetical protein	ATI16967.1	93%	$5e^{-28}$	44.20%
11	6780	9431	RNA polymerase	ATW57716.1	100%	0.0	99.21%
12	9747	9995	Inhibitor of dGTPase	AUV62633.1	100%	$1e^{-28}$	63.53%
13	10074	11120	DNA ligase	AUV62634.1	100%	0.0	94.25%
14	11264	11566	hypothetical protein	AUV62635.1	99%	$2e^{-64}$	98.99%
15	11563	11844	nucleotide kinase	AYD80135.1	92%	$2e^{-52}$	94.19%
16	11795	11971	hypothetical protein	YP_002003946.1	63%	$7e^{-19}$	97.30%

17	11973	12167	RNA polymerase inhibitor	AUV62637.1	100%	$1e^{-39}$	100.00%
18	12233	12934	single-stranded DNA-binding protein	ATW57727.1	100%	$7e^{-168}$	99.15%
19	13390	13845	endolysin	AUV62639.1	100%	$6e^{-110}$	100%
20	13906	14394	hypothetical protein	AVJ51901.1	99%	$4e^{-101}$	83.85%
21	14372	16066	DNA primase/helicase	NP_041975.1	100%	0.0	92.40%
22	16113	16325	hypothetical protein	NP_041979.1	100%	$2e^{-40}$	98.57%
23	16345	16614	inhibitor of toxin/antitoxin system	AYD80143.1	100%	$3e^{-55}$	96.63%
24	16559	17095	hypothetical protein	AYD82935.1	91%	$8e^{-107}$	94.48%
25	17114	19096	DNA polymerase	NP_041982.1	98%	0.0	99.23%
26	19158	19553	endonuclease	YP_002003959.1	100%	$2e^{-91}$	98.47%
27	19849	20148	HNS binding protein	ATW57736.1	100%	$4e^{-63}$	96.78%
28	20148	20357	HNS binding protein	YP_009198317.1	100%	$2e^{-37}$	84.06%
29	20267	20515	Putative RecBCD inhibitor	AYD80147.1	63%	$5e^{-29}$	98.08%
30	20502	21404	exonuclease	ATW57739.1	100%	0.0	99.00%
31	21603	21857	hypothetical protein	ATW57741.1	100%	$4e^{-56}$	100%
32	21844	22128	hypothetical protein	AUV62648.1	100%	$4e^{-59}$	97.87%
33	22128	22529	hypothetical protein	AUV62649.1	100%	$6e^{-92}$	96.99%
34	22533	22847	hypothetical protein	AYD82945.1	100%	$6e^{-44}$	89.42%
35	22862	24472	head-to-tail connector	ATW57745.1	100%	0.0	99.25%
36	24572	25495	scaffold protein	AUV62653.1	100%	0.0	98.70%
37	25594	26634	minor capsid protein	NP_041997.1	99%	0.0	93.02%
38	26634	26789	minor capsid protein	AUV62655.1	100%	$7e^{-23}$	100.00%
39	26858	27448	tail tubular protein A	WP_015971119.1	100%	$3e^{-140}$	98.47%

40	27469	29856	tail tubular protein B	YP_009291512.1	100%	0.0	92.08%
41	29936	30352	putative scaffold protein	AUV62658.1	100%	5e^{-98}	98.55%
42	30355	30933	internal virion protein	AUV62659.1	100%	1e^{-118}	92.86%
43	30940	32448	internal virion protein	AYD80207.1	100%	0.0	91.25%

Table S6: ORF comparison and annotation of phage HZ2R8

ORF	Start	Stop	Description	ID	Query cover	Evalue	Identify
1	61	264	class II holin	WP_113998049.1	100%	3e^{-40}	100%
2	264	539	DNA packaging protein	WP_113998050.1	97%	2e^{-57}	98.88%
3	634	1080	endopeptidase	ATW57758.1	100%	1e^{-99}	96.62%
4	1077	2849	DNA packaging protein	YP_009152457.1	100%	0.0	98.47%
5	2887	3330	hypothetical protein	AYD82961.1	100%	1e^{-102}	95.92%
6	5252	5407	hypothetical protein	ATW57710.1	100%	6e^{-30}	100%
7	6424	7485	protein kinase	NP_041959.1	100%	0.0	86.40%
8	7555	10206	RNA polymerase	ATW57716.1	100%	0.0	99.09%
9	10438	10779	inhibitor of dGTPase	ATW57718.1	75%	3e^{-52}	92.94%
10	10860	11882	DNA ligase	AYD82924.1	100%	0.0	94.41%
11	12011	12325	hypothetical protein	AYD80134.1	82%	8e^{-53}	98.84%
12	12325	12864	nucleotide kinase	AYD80135.1	99%	4e^{-124}	95.51%
13	12978	13172	RNA polymerase inhibitor	ATW57726.1	100%	5e^{-39}	98.44%
14	13238	13936	single-stranded DNA-binding protein	YP_009152474.1	100%	6e^{-167}	98.71%
15	14392	14847	endoysin	ATW57729.1	100%	8e^{-109}	98.68%
16	14919	16619	DNA primase/helicase	AYD82932.1	100%	0.0	99.82%
17	16666	16878	hypothetical protein	ATW57731.1	100%	4e^{-40}	98.57%

18	16898	17167	inhibitor of toxin/antitoxin system	AYD80143.1	100%	$3e^{-56}$	97.75%
19	17680	19794	DNA-directed DNA polymerase	AYD80145.1	100%	0.0	99.43%
20	19926	20117	hypothetical protein	YP_009291500.1	93%	$1e^{-26}$	80.00%
21	20319	20477	inihibitor of recBCD nuclease	YP_009152484.1	98%	$7e^{-29}$	100%
22	20464	21366	exonuclease	AYD82940.1	100%	0.0	99.33%
23	21565	21819	hypothetical protein	NP_848289.1	100%	$1e^{-53}$	95.24%
24	21806	22090	hypothetical protein	AYD80150.1	100%	$1e^{-58}$	96.81%
25	22090	22491	hypothetical protein	WP_015979372.1	100%	$7e^{-91}$	96.24%
26	22495	22803	hypothetical protein	YP_009152490.1	77%	$1e^{-38}$	92.41%
27	22818	23210	hypothetical protein	AYD80198.1	100%	$7e^{-90}$	97.69%
28	23195	24820	Head-to-tail connector protein	AYD80199.1	100%	0.0	97.97%
29	24911	25849	capsid assembly scaffolding protein	YP_009291508.1	98%	0.0	98.05%
30	25948	26982	minor capsid protein	AGB07356.1	99%	0.0	96.49%
31	26982	27137	minor capsid protein	QBG78760.1	96%	$2e^{-13}$	78.00%
32	27206	27796	tail tubular protein	WP_015971119.1	100%	$5e^{-140}$	97.96%
33	27817	30204	tail tubular protein	YP_009291512.1	100%	0.0	91.82%
34	30284	30700	putative scaffold protein	AXC37103.1	100%	5e-98	98.55%
35	30704	31294	internal virion protein	WP_113998045.1	100%	$1e^{-128}$	92.86%
36	31301	33544	internal virion protein	WP_113998046.1	100%	0.0	97.19%
37	33570	37526	internal virion protein	AYD80162.1	100%	0.0	93.40%
38	37599	39434	tail fiber protein	QBG78753.1	100%	0.0	66.14%

Table S7: Three T7-like *E. coil* phages with high total scores

E. coli Bacteriophage	Accession	Time	Location
HZP2	MK542821	2019.2.19	China.Huizhou
HZ2R8	MG832642	2018.1.22	China.Huizhou
T7	AY264774	2003.3.28	America
64795_ec1	KU927499	2016.3.11	Italy. Portici
EG1	MG488277	2017.11.9	China.Nanjing

Reference

Aminov, R. I., N. Garrigues-Jeanjean, and R. I. Mackie. 2001. 'Molecular ecology of tetracycline resistance: development and validation of primers for detection of tetracycline resistance genes encoding ribosomal protection proteins', *Appl Environ Microbiol*, 67: 22-32.

Calero-Caceres, W., A. Melgarejo, M. Colomer-Lluch, C. Stoll, F. Lucena, J. Jofre, and M. Muniesa. 2014. 'Sludge as a potential important source of antibiotic resistance genes in both the bacterial and bacteriophage fractions', *Environ Sci Technol*, 48: 7602-11.

Cattoir, V., L. Poirel, V. Rotimi, C. J. Soussy, and P. Nordmann. 2007. 'Multiplex PCR for detection of plasmid-mediated quinolone resistance qnr genes in ESBL-producing enterobacterial isolates', *J Antimicrob Chemother*, 60: 394-7.

CLSI. 2018. 'Performance standards for antimicrobial susceptibility testing', *Clinical and Laboratory Standards Institute*.

Guo, M. T., Q. B. Yuan, and J. Yang. 2013. 'Ultraviolet reduction of erythromycin and tetracycline resistant heterotrophic bacteria and their resistance genes in municipal wastewater', *Chemosphere*, 93: 2864-8.

Pei, R., S. C. Kim, K. H. Carlson, and A. Pruden. 2006. 'Effect of river landscape on the sediment concentrations of antibiotics and corresponding antibiotic resistance genes (ARG)', *Water Res*, 40: 2427-35.

Xi, C., Y. Zhang, C. F. Marrs, W. Ye, C. Simon, B. Foxman, and J. Nriagu. 2009. 'Prevalence of antibiotic resistance in drinking water treatment and distribution systems', *Appl Environ Microbiol*, 75: 5714-8.
